# Violência doméstica e constipação intestinal: uma revisão integrativa da literatura

**DOI:** 10.26633/RPSP.2017.19

**Published:** 2017-03-03

**Authors:** Ana Lúcia Couto Coronel, Helena Terezinha Hubert Silva

**Affiliations:** 1 Universidade Federal de Ciências da Saúde de Porto Alegre Programa de Pós-Graduação em Ensino na Saúde Porto Alegre (RS) Brasil Universidade Federal de Ciências da Saúde de Porto Alegre, Programa de Pós-Graduação em Ensino na Saúde, Porto Alegre (RS), Brasil.; 2 Universidade Federal de Ciências da Saúde de Porto Alegre Departamento de Patologia e Medicina Legal e Programa de Pós-Graduação em Ensino na Saúde Porto Alegre (RS) Brasil Universidade Federal de Ciências da Saúde de Porto Alegre, Departamento de Patologia e Medicina Legal e Programa de Pós-Graduação em Ensino na Saúde, Porto Alegre (RS), Brasil.

**Keywords:** Violência doméstica, constipação intestinal, abuso físico, Domestic violence, constipation, physical abuse

## Abstract

**Objetivo.:**

*Buscar evidências na literatura sobre a relação entre violência doméstica e constipação intestinal*.

**Metodologia.:**

*Foi realizada uma revisão integrativa, baseada no método preconizado em seis etapas e construída conforme a metodologia PRISMA* (Preferred Reporting Items for Systematic Reviews and Meta-Analyses)*. Foram examinados artigos publicados entre 2005 e 2015 que relacionassem violência doméstica e constipação intestinal. As buscas ocorreram em setembro e outubro de 2015 nas bases de dados PubMed, MEDLINE, Scopus e Web of Science. A seleção compreendeu três etapas: busca, pré-seleção e inclusão de artigos*.

**Resultados.:**

*Dos 177 artigos inicialmente identificados, foram selecionados 11. Dos 11 selecionados, sete eram quantitativos, três eram qualitativos e um era misto. Quatro enfocaram crianças e adolescentes, dois investigaram o conhecimento médico sobre a relação entre violência doméstica e constipação intestinal, um revisou distúrbios digestivos em idosos, um avaliou os resultados do* biofeedback *em constipados com e sem histórico de violência, um avaliou disfunção evacuatória e relacionou o resultado de defecografias com histórico de violência doméstica e dois estudos eram relatos de especialistas. Três estudos apresentaram nível de evidência 1B e grau de recomendação A. Todos os estudos detectaram relação entre violência doméstica e constipação intestinal*.

**Conclusão.:**

*Os resultados desta revisão indicam que existe correlação entre violência doméstica e constipação intestinal. É recomendável a investigação dessa relação nas práticas em saúde*.

## Abstract (en)

**Objective:**

To search the literature for evidence of a relationship between domestic violence and constipation.

**Methods:**

An integrative review was conducted using the six-step approach and the Preferred Reporting Items for Systematic Reviews and Meta-Analyses (PRISMA). We examined articles published between 2005 and 2015 investigating the correlation between domestic violence and constipation. The search was performed in September and October of 2015 in PubMed, MEDLINE, Scopus, and Web of Science. The selection of articles was performed in three stages: search, pre-selection, and inclusion.

**Results:**

Of 177 articles identified, 11 were included in the review. Of these 11 articles, seven were quantitative, three were qualitative, and one was mixed. Four articles focused on children and adolescents, two investigated medical knowledge regarding the relationship between domestic violence and constipation, one reviewed digestive disorders in the elderly, one evaluated the results of biofeedback for the treatment of constipated individuals with or without a history of violence, and one evaluated evacuatory disorders and correlated the result of defecography with history of domestic violence. Finally, two studies were specialist reports. Level of evidence was 1B and recommendation grade A in three studies. All studies detected a relationship between domestic violence and intestinal constipation.

**Conclusion:**

The results of the present review support a correlation between domestic violence and intestinal constipation. The investigation of this relationship is recommended in clinical practice.

Em 1996, a Organização Mundial da Saúde (OMS) reconheceu a violência como um grave problema de saúde pública. Em 2002, o lançamento do primeiro Relatório Mundial sobre Violência e Saúde motivou muitos países a discutirem o assunto e desenvolverem políticas públicas para o enfrentamento da violência (1). Em 2014, em seu Relatório Mundial sobre Prevenção à Violência, que traz dados de 133 países e é o primeiro relato desse tipo a avaliar o combate à violência interpessoal, a OMS revelou lacunas nas medidas preventivas adotadas. Também reconheceu que as consequências da violência não são apenas imediatas, como a morte e a mutilação, mas que se estendem a longo prazo, repercutindo de diversos modos sobre a saúde das vítimas (2).

Mulheres, crianças, adolescentes, idosos e deficientes são os grupos populacionais mais vulneráveis à violência doméstica, com consequências imediatas e tardias reconhecidas nas esferas psicológica, emocional e física (3). Os tipos de violência mais referidos são o abuso físico, psicológico e sexual (4–6). Ao longo dos anos, estudos vêm apontando prováveis efeitos da violência doméstica sobre o aparelho digestivo e recomendam essa busca na investigação clínica. Um estudo recente recomenda a investigação da violência sobretudo em portadores de doenças funcionais do aparelho digestivo, tais como a síndrome do intestino irritável, a dispepsia funcional, a dor abdominal funcional e a constipação intestinal funcional (7).

A constipação intestinal já foi apontada como a queixa mais frequente entre as pessoas que buscam atendimento médico por problemas digestivos (8). Entre 2001 e 2004, foi a causa de cerca de 8 milhões de consultas ambulatoriais nos Estados Unidos (9). Um estudo realizado no Brasil na cidade de Pelotas, estado do Rio Grande do Sul, avaliou a constipação intestinal na população geral e também demonstrou uma prevalência elevada, em torno de 27% (10). Entretanto, o histórico de violência doméstica não foi pesquisado nesses indivíduos.

Semelhante à violência doméstica, a constipação intestinal atinge todos os grupos populacionais, sendo predominante em mulheres, idosos e populações com baixas condições socioeconômicas. Os indivíduos afetados têm sua qualidade de vida comprometida, além de um elevado grau de insatisfação quanto aos resultados dos tratamentos (11). Contudo, mesmo naqueles indivíduos que preenchem os critérios para diagnóstico de constipação intestinal funcional, o histórico de violência doméstica não é rotineiramente investigado (12).

Nesse contexto, cabe indagar se de fato existe uma relação entre esses fenômenos. Assim, o objetivo do presente estudo foi realizar uma revisão da literatura em busca de evidências sobre a relação entre violência doméstica e constipação intestinal.

## MATERIAIS E MÉTODOS

O método escolhido para o presente estudo foi a revisão integrativa da literatura (13), seguindo o procedimento preconizado de seis etapas (14): identificação do tema e seleção da hipótese, estabelecimento da estratégia de pesquisa, definição e coleta de dados, análise dos dados coletados, interpretação e apresentação dos resultados. A revisão do processo baseou-se nas recomendações da lista de conferência *Preferred Reporting Items for Systematic Reviews and Meta-Analyses* (PRISMA) (15).

A pesquisa de artigos foi feita por um investigador independente, através do portal de periódicos da Coordenação de Aperfeiçoamento de Pessoal de Nível Superior (CAPES), com acesso às seguintes bases de dados eletrônicas: PubMed, *Medical Literature Analysis and Retrieval System Online* (MEDLINE), Scopus e Web of Science. Foram, também, realizadas buscas manuais nas listas de referências dos artigos selecionados. As buscas ocorreram em setembro e outubro de 2015. Foram utilizados os seguintes descritores em ciências da saúde (DeCS): *constipation (and): domestic violence, abuse, neglect*. Optou-se pela utilização específica do descritor *domestic violence*, ao invés do termo genérico *violence*, tendo em vista o escopo da pesquisa. Ao utilizar o descritor *abuse*, foi possível abranger abuso físico, psicológico e sexual em adultos de ambos os sexos e de todas as idades, assim como em crianças e adolescentes. Não foi feita uma distinção entre constipação primária ou secundária e seus subtipos, pois a maioria dos estudos encontrados não fazia essa diferenciação.

Foram aplicados filtros de busca conforme os critérios de inclusão e exclusão determinados. Os critérios de inclusão adotados foram: ser artigo científico, em língua portuguesa, inglesa ou espanhola, publicado em periódicos revisados por pares, entre os anos de 2005 e 2015, oferecer texto para leitura na íntegra e tópico que relacionasse violência doméstica e constipação intestinal. Foram excluídos artigos duplicados, revisões sistemáticas, teses, dissertações, editoriais, cartas e similares.

**FIGURA 1.**
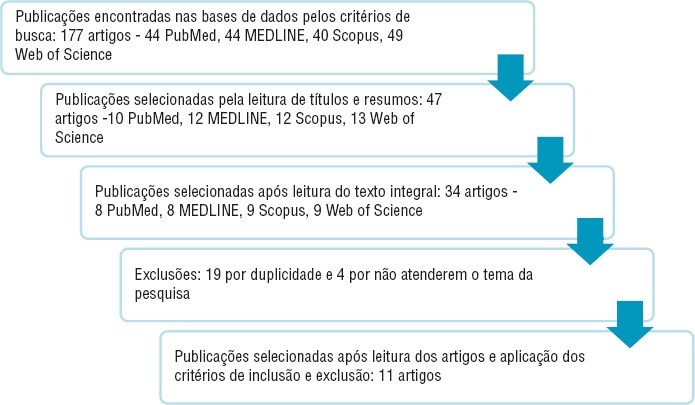
Fluxograma da seleção de estudos sobre violência doméstica e constipação publicados entre 2005 e 2015

A pré-seleção de artigos foi feita pela leitura preliminar de títulos e resumos. Os estudos pré-selecionados foram lidos na íntegra para seleção final dos artigos para análise. Esta fase está representada na figura 1.

Os dados dos artigos selecionados foram registrados individualmente, por ordem de data de publicação, em uma matriz de coleta de dados, com destaque para tipologia, objetivo, metodologia, resultados, conclusão, nível de evidência e grau de recomendação de cada estudo (tabela 1). Os artigos foram classificados quanto ao nível de evidência (1A, 1B, 1C, 2A, 2B, 2C, 3A, 3B, 4 ou 5, sendo 1A o nível de evidência mais robusto, com maior aplicabilidade clínica) e ao grau de recomendação (A, B, C ou D, sendo os estudos com nível de evidência mais alto agrupados na categoria A, que reflete maior confiabilidade dos dados) (16).

## RESULTADOS

Foram identificados 177 artigos nas bases de dados pesquisadas através das estratégias de busca (figura 1). Após leitura dos títulos e resumos, 47 artigos foram considerados potencialmente elegíveis para inclusão no estudo e foram recuperados para leitura na íntegra. Após a leitura completa, 11 artigos foram selecionados mediante aplicação dos critérios de inclusão e exclusão estabelecidos, sendo três do tipo qualitativo (sendo o termo “artigo qualitativo” definido como qualquer pesquisa que empregue informação não numérica para explorar características individuais ou de grupo e que produz achados não acessíveis por procedimentos estatísticos ou outro meio quantitativo) (17), sete do tipo quantitativo e um do tipo misto. Trinta e um artigos foram excluídos por duplicidade, ou seja, o mesmo estudo ocorreu em mais de uma base de dados. Cinco artigos, que relacionavam distúrbios digestivos e violência doméstica em seus resumos, foram excluídos por não citarem especificamente constipação intestinal no texto.

**TABELA 1.** Matriz de coleta de dados sobre constipação e violência doméstica em artigos publicados entre 2005 e 2015

Artigo/data (ref)	Tipo^a^	Objetivo	Métodos	Resultados	Conclusão	NE/GR^b^
Aggressiveness and hostility in the family environment and chronic constipation in children: 2008 (18)	QT	Comparar aspectos emocionais desencadeados pela relação familiar de crianças com e sem constipação.	14 crianças constipadas, de 7 a 12 anos, foram comparadas a um grupo controle não constipado comrelação a aspectos emocionais, comportamentais, dedesenvolvimento e familiares. As mães responderam questionários não estruturados sobre aspectos emocionais de seusfilhos e as crianças foram estimuladas a contar histórias a partir de um painel com teste de percepção temática para crianças.	O grupo de crianças constipadas apresentou mais distúrbios emocionais do que o grupo controle, com diferenças estatisticamente significativas; as mães das crianças constipadas tinham mais aspectos disfóricos e as famílias tinham mais agressividade e punições (P < 0,05), retratadas nas histórias das crianças.	A constipação nas crianças estava associada a agressividade e ambiente familiar hostil, intolerância a frustração, dificuldade na escola e depressão e ansiedade maternas. Mais estudos são necessários para aprofundar o conhecimento sobre os fatores biopsicossociais relacionados à constipação, para um atendimento integral aos pacientes.	2B/C
Conservative and behavioral management of constipation: 2009 (27)	NO	Contribuir para o entendimento e manejo das alterações psicológicas e evacuatórias em pacientes com histórico de abuso.	Não relatado.	O abuso físico e sexual é mais frequente em pessoas com distúrbios gastrointestinais do que na população em geral (40-50% vs. 20%), é mais fortemente associado com queixas digestivas baixas e é mais comumente encontrado em pessoas constipadas.	Pacientes com distúrbios digestivos refratários devem ser investigados quanto ao histórico de abuso. Quando o abuso for identificado, os pacientes devem ser encaminhados para tratamento psicológico, embora não existam estudos controlados mostrando os benefícios dessa abordagem.	5/D
Association between constipation and stressful life events in a cohort of Sri Lankan children and adolescents: 2009 (19)	QT	Avaliar a associação entre constipação e estresse emocional em crianças e adolescentes.	Foram avaliados, prospectivamente, 2699 escolares de 10 a 16 anos de quatro escolas selecionadas randomicamente. Foram submetidas a um questionário estruturado autoaplicado, que incluía dados demográficos, histórico de eventos estressantes nos últimos 3 meses e critérios de Roma III e escala de Bristol para constipação intestinal.	416 indivíduos (15,4%) tinham constipação. Constipação foi maior naqueles expostos a estresse emocional (OR 2,52; P <0,0001).	Constipação foi significativamente maior em crianças e adolescentes expostos a estresse emocional (inclusive violência doméstica).	1B/A
Abuse, trauma, and GI illness: is there a link?: 2011 (12)	NO	Descrever a compreensão do autor quanto ao efeito do abuso e trauma na doença gastrointestinal, fornecer a justificativa científica para essa associação, oferecer orientações a respeito de quando e como obter informações sobre essa questão e orientar para implementar o cuidado adequado para o paciente.	Não relatado.	O papel do abuso e do trauma na história dos pacientes, em particular com doença digestiva, e a base científica para essa associação têm evoluído ao longo das últimas 3 décadas.	A história de abuso pode ser altamente prevalente entre os pacientes e pode ter um importante impacto sobre o cuidado clínico que precisa ser oferecido. Compreender essa associação, saber quando perguntar sobre história de abuso e saber como prestar cuidados direcionados são obrigações médicas, particularmente para doentes com sintomas gastrointestinais mais severos.	5/D
Gastrointestinal issues in the older female patient: 2011 (24)	NO	Revisar questões gastrointestinais que comumente afligem a população idosa.	Não relatado.	Refere estudos que apontam para uma incidência de 22 a 48% de abuso sexual e 31 a 74% de abuso físico em adultos constipados.	A terapia deve ser individualizada, começando por uma dieta rica em fibras, atividade física, líquidos laxativos, antagonistas de opioides, *biofeedback* e cirurgia para casos refratários. Através do reconhecimento dos sintomas, pode-se iniciar tratamento específico.	5/D
Sexual abuse history in GI illness, how do gastroenterologists deal with it?: 2012 (22)	QT	Avaliar se os gastroenterologistas abordam abuso sexual em sua prática diária e avaliar os seus conhecimentos quanto ao alcance do abuso sexual na doença gastrointestinal, inclusive para constipação intestinal.	402 questionários foram enviados por correio eletrônico para os membros da Sociedade Holandesa de Gastroenterologistas, incluindo questões sobre a prática clínica referente à investigação e conhecimento sobre a interação entre abuso sexual e doenças digestivas.	169 questionários foram analisados: 4,7% referiram que questionavam mulheres e 0,6% homens quanto ao abuso sexual; antes de colonoscopia, essas taxas caíam para 2,4% e 0,6%. Quando havia queixas específicas, como dor abdominal, incontinência fecal e constipação, os índices aumentavam para 71,4% e 31,3%, respectivamente.	Gastroenterologistas não costumam perguntar sobre a história de abuso sexual e raramente perguntam sobre isso antes de realizar a colonoscopia. Há uma necessidade de melhorar a formação quanto a habilidades e conhecimentos para lidar com abuso sexual.	3B/B
A randomized controlled trial of anorectal biofeedback for constipation: 2012 (25)	QT	Avaliar a resposta ao tratamento de *biofeedback* para constipação intestinal e a influência do histórico de abuso físico e sexual na resposta ao tratamento.	21 pacientes com discinesia do assoalho pélvico foram randomizados para o *biofeedback*: grupo A recebeu tratamento para assoalho pélvico e grupo B recebeu tratamento para outros grupos musculares; todos foram submetidos aos questionários (CSI, IBS-QOL, SF-36, PCS, MCS e Trauma History Questionnaire), antes e após o tratamento.^c^	No grupo não abusado os escores CSI totais caíram 29%. No grupo abusado, caíram 12,6%; aqueles sem histórico de abuso físico/sexual na infância relataram menos constipação, em média, do que aqueles com histórico de abuso. Na escala CSI-defecação obstrutiva, os escores do grupo sem abuso caíram 28,8%, e os escores do grupo abusado caíram 22,3%. Finalmente, no MCS, o grupo sem abuso melhorou 12,3% em saúde mental vs. grupo com histórico de abuso, que mostrou diminuição de 21,6%.^c^	Os dados sugerem que uma história de abuso sexual e físico na infância pode estar associada com melhoria diminuída na gravidade da dor e da qualidade de saúde mental, após *biofeedback*.	2B/B
What do pediatrics residents know about the psychological factors in constipation?: 2013 (23)	QT	Avaliar o conhecimento dos residentes de pediatria de Salvador, Bahia, sobre a relação entre a constipação intestinal e fatores psicológicos.	Aplicação de um questionário a 42 médicos residentes de pediatria, dividido em cinco domínios, sendo nove questões objetivas e 12 subjetivas, relacionadas ao tema de pesquisa.	45,2% consideraram de 6 a 12 meses o tempo necessário para tratamento da constipação intestinal; 33,3% consideraram 6 meses e 1, 2 anos. A dieta foi apontada como causa por 95,2%; 38% referiram fatores psicológicos (abuso, conflito familiar, agressão) como possível causa e 23,8%, como possível fator desencadeante.	O conhecimento dos entrevistados sobre fatores psicológicos e trauma envolvidos na constipação intestinal é inadequado. Énecessária uma abordagem mais profunda e multidisciplinar, com educação médica continuada sobre constipação intestinal.	2B/B
A prospective study evaluating emotional disturbance in subjects undergoing defecating proctography: 2013 (26)	QT	Avaliar a prevalência de doenças psiquiátricas em indivíduos com distúrbio evacuatório através de defecografia e questionários validados.	Pacientes encaminhados para defecografia por distúrbio evacuatório responderam questionários validados (HADS, STAI, PHQ-15, PTSD), questionário de Roma III e questionário validado para abuso físico e sexual. Foram divididos em dois grupos: com e sem alterações na defecografia.^d^	45 indivíduos foram avaliados: 20 (44,4%) com defecografia normal: a incidência de abuso sexual foi maior no grupo com defecografia normal (P < 0,036); nos questionários HADS, STAI, PHQ-15 e PTSD, os escores do grupo com defecografia normal foram maiores: *P* = 0,04- ansiedade/0,01-vdepressão, *P* = 0,027, *P* = 0,038, *P* = 0,045.^d^	Significativo aumento de doenças psiquiátricas em indivíduos com evacuação alterada e defecografia normal. Esses indivíduos devem ser investigados para histórico de abuso sexual e desordens psiquiátricas.	2B/B
Association between child maltreatment and constipation: a school-based survey using Rome III criteria: 2014 (20)	QT	Avaliar a relação entre abuso infantil e constipação em escolares do Distrito de Gampaha, Sri Lanka,	Foram selecionadas, randomicamente, quatro escolas, entre 427. Avaliados 1792 estudantes com idade de 13 a 17 anos através de um questionário estruturado autoadministrado, incluindo os critérios de Roma III.	975 (54,4%) com idade média de 14,4 anos; 138 (7,7%) constipados; 438 (24,4%) com abuso físico, 396 (22,1%) com abuso emocional; e 51 (2,8%) com abuso sexual. Nos constipados a incidência foi maior para abuso físico = 41,6 vs. 23,2 - P < 0,0001, abuso emocional = 40,9 vs. 20,8 - P < 0,0001, abuso sexual = 5,8 vs. 2,6 - *P* < 0,03. Somatização maior em constipados abusados; abusados tinham percepção de maior severidade dos sintomas.	A constipação na infância e adolescência mostra associação significativa com histórico de abuso físico, emocional e sexual. Quando abusados, crianças e adolescentes constipados apresentam mais queixas físicas e intestinais.	1b/A
Association between functional gastrointestinal diseases and exposure to abuse in teenagers: 2014 (21)	QT	Identificar a relação entre abuso e doenças gastrointestinais funcionais, com constipação predominante (DGIF-DP).	Os adolescentes foram recrutados de quatro escolas selecionadas aleatoriamente na província ocidental de Sri Lanka. Os dados foram coletados por questionário autoadministrado validado. Doenças gastrointestinais funcionais foram diagnosticadas usando critérios de Roma III.	1850 adolescentes com 13 a 18 anos foram incluídos; 305 (16,5%) tiveram doenças gastrointestinais funcionais, que foram significativamente maiores em pessoas expostas a abuso sexual (34,0%), emocional (25,0%) e físico (20,2%) do que em não expostos (13,0%, *P*< 0,001). Aqueles com doenças gastrointestinais funcionais expostos a abusos tiveram pontuação maior na gravidade dos sintomas intestinais (30,8% vs. 24,7% em não abusados, *P*<0,05).	Este estudo destaca a importância de identificar a exposição a abusos no manejo de adolescentes com doenças gastrointestinais funcionais com constipação predominante.	1b/A

a QT, quantitativo; NO, não original.

b NE, nível de evidência; GR, grau de recomendação.

c CSI, Constipation Severity Instrument; IBS-QOL, Irritable Bowel Syndrome Quality of Life Scale; SF-36, Medical Outcomes Study Short Form-36; PCS, Physical Health Composite Scale; MCS, Mental Health Composite Scale; Trauma History Questionnaire.

d HADS, Hospital Anxiety and Depression Scale; STAI, State Trait Anxiety Inventory; PHQ-15, Patient Health Questionnaire 15-item somatic symptom severity scale, validated questionnaires for sexual or physical abuse; PTSD, Post-traumatic Stress Disorder questionnaire.

Com relação à população estudada, quatro estudos estão relacionados à violência doméstica em crianças ou adolescentes constipados (18–21). Dois estudos buscaram determinar o conhecimento médico quanto à associação entre doenças gastrointestinais e histórico de violência doméstica (22, 23). Um estudo revisou doenças gastrointestinais e suas causas em idosos (24). Um estudo avaliou o histórico de abuso e alterações psicológicas em adultos com disfunção evacuatória (25). Um estudo comparou o resultado de defecografias em adultos constipados com e sem histórico de abuso (26) e dois estudos apresentaram relatos de experiência e opiniões de especialistas (12, 27).

Quanto às intervenções realizadas, três artigos apresentaram revisões não sistemáticas e, portanto, não houve intervenção ou descrição de metodologia (12, 24, 27). Três estudos utilizaram questionários estruturados, autoadministrados (19–21), e um utilizou um questionário não estruturado e um painel com teste de percepção temática para crianças (18).

Um estudo avaliou a resposta a *biofeedback* em indivíduos com disfunção evacuatória em dois grupos randomizados (com e sem tratamento) e aplicou questionários validados para avaliar a constipação intestinal, síndrome do intestino irritável e qualidade de vida, alterações psicológicas e histórico de trauma. Os questionários utilizados foram: *Constipation Severity Instrument* (CSI), *Irritable Bowel Syndrome Quality of Life Scale* (IBS-QOL), *Medical Outcomes Study Short Form-36* (SF-36), *Physical Health Composite Scale* (PCS), *Mental Health Composite Scale* (MCS) e *Trauma History Questionnaire* (25). Os dois estudos que avaliaram o conhecimento médico utilizaram questionários semiestruturados envolvendo perguntas sobre a prática clínica e o conhecimento sobre a relação entre abuso e doenças gastrointestinais (22, 23). Um estudo coletou o histórico de abuso físico, psicológico e sexual através de questionários validados e caracterizou a constipação intestinal pelos critérios de Roma III, comparando os resultados da defecografia de pessoas constipadas com e sem histórico de violência. Os questionários utilizados foram: *Hospital Anxiety and Depression Scale* (HADS), *State-Trait Anxiety Inventory* (STAI)*, Patient Health Questionnaire 15-Item Somatic Symptom Severity Scale* (PHQ-15)*,* questionários validados que medem abuso físico e sexual e um instrumento de avaliação de síndrome do estresse pós-traumático (26).

Embora tenham sido buscados artigos publicados entre 2005 e 2015, não foram encontrados estudos que atendessem aos critérios de inclusão entre 2005 e 2007. Entre os artigos incluídos, um artigo foi publicado em 2008 (19), dois em 2009 (19, 27), dois em 2011 (12, 24), dois em 2012 (22, 25), dois em 2013 (23, 26) e dois em 2014 (20, 21).

Quanto ao nível de evidência e grau de recomendação, três estudos de coorte foram classificados como 1B com grau de recomendação A (19-21); três foram classificados como 2B com grau de recomendação B (23, 25, 26); um foi classificado como 2B com grau de recomendação C (18); um foi classificado como 3B com grau de recomendação B (22); e três estudos não originais (um de revisão narrativa e dois de opinião e relato de experiência) foram classificados como nível de evidência 5 e grau de recomendação D (12, 24, 27). O tipo mais citado de violência doméstica foi o abuso sexual.

Quatro estudos (18–21) referiram em suas conclusões a associação entre violência doméstica e constipação intestinal (36,4%). Dois estudos, que analisaram o conhecimento médico quanto à interação entre violência doméstica e constipação intestinal, reconheceram essa associação e apontaram para a necessidade de maior capacitação profissional nessa questão (22, 23).

Um estudo concluiu que, em indivíduos com disfunção evacuatória e história de violência, a defecografia tem maior índice de normalidade, apesar da sintomatologia (26). Outro estudo concluiu que indivíduos constipados com histórico de abuso têm pior resposta ao tratamento da constipação intestinal por *biofeedback* (25).

Dois estudos que apresentam o relato de especialistas concluíram que a ocorrência de violência doméstica deve ser investigada em pessoas com queixas digestivas, especialmente os casos refratários e severos (12, 27). Por fim, o estudo relacionado aos distúrbios gastrointestinais em idosos não referiu a associação com violência doméstica na conclusão, apesar de abordá-la durante a revisão (24).

## DISCUSSÃO

Antes de 2005, revisões de literatura, assim como artigos teóricos e empíricos, já vinham buscando a relação entre o histórico de violência e as doenças gastrointestinais. Esses trabalhos concluíram que essa inter-relação existe e recomendaram a sua inclusão na investigação clínica (5, 6, 28). Entretanto, essas recomendações ainda não fazem parte da rotina assistencial (22, 23). Os estudos aqui integrados continuam a apontar na mesma direção. Apesar dos diferentes níveis de evidência, existe convergência entre as recomendações de diferentes estudos.

Sabe-se que um dos fatores de risco para tornar-se um agressor é ter sido vítima de agressão, perpetuando o ciclo da violência (29). Os resultados encontrados em crianças e adolescentes constipados sugerem a necessidade de considerar a hipótese de violência doméstica e incluir esse questionamento na avaliação (18–21). A intervenção precoce, além de reduzir os danos decorrentes da violência, tem um importante papel preventivo. Em contrapartida, a falta de conhecimento e o despreparo dos médicos avaliados em alguns estudos demonstram que é preciso maior capacitação profissional para essa abordagem (22, 23). É possível que, além da necessidade de incorporar essa discussão na formação acadêmica dos profissionais da saúde, tabus sociais e culturais também devam ser vencidos. As revisões e relatos de especialistas reafirmam essa necessidade, além de ressaltar o papel fundamental da relação de confiança entre médico e paciente (12, 27).

As observações realizadas nos estudos que avaliaram resultados terapêuticos (25) e resultados de exames complementares (26) em adultos constipados apontam para a importância de considerar a hipótese de violência doméstica frente a resultados terapêuticos e diagnósticos insatisfatórios ou controversos. O único estudo com idosos (24) referiu superficialmente a relação entre constipação intestinal, abuso e negligência, deixando uma importante lacuna sobre essa questão. Embora o interesse pelo tema da violência contra o idoso e suas consequências tenha crescido nos últimos anos, muitas questões ainda precisam ser investigadas (30).

O número reduzido de estudos encontrados alerta principalmente para a necessidade de aprofundar as discussões sobre o tema. Embora estudos com maior rigor metodológico sejam um desafio para os pesquisadores, a prática baseada em evidências tem-se mostrado um caminho seguro para as tomadas de decisão em saúde (31).

O presente estudo tem limitações que merecem atenção. As revisões integrativas têm o potencial de encontrar maiores informações sobre o estado da arte de um determinado tema, pois reúnem várias perspectivas em diferentes metodologias. Entretanto, pela complexidade que representa buscar, integrar e analisar métodos diversos, pode-se perder em rigor e incorrer em vieses de interpretação, muitas vezes deixando de incluir importantes estudos na área (14). Outras limitações são o número reduzido de artigos encontrados e a diversidade das populações estudadas, que dificultam a extrapolação dos resultados. Entretanto, apesar dessas limitações, acreditamos que este estudo tenha alcançado o objetivo de encontrar evidências sobre o estado atual das investigações acerca da relação entre violência doméstica e constipação intestinal.

### Considerações finais

A hipótese da existência de uma inter-relação entre violência doméstica e constipação intestinal foi confirmada pela maioria dos estudos encontrados, especialmente em crianças e adolescentes. Como foi descrito, a violência doméstica tem amplas repercussões sociais, humanas e políticas, ocultando-se nas mais diversas formas e causando as mais variadas consequências. Seus reflexos sobre a saúde estão bem documentados e é necessário abordar o tema nas avaliações clínicas.

Concluímos que há evidências da relação entre violência doméstica e constipação intestinal e recomendamos a busca cautelosa dessa relação nas práticas em saúde. Esperamos que essa busca gere novos estudos com níveis de evidência e graus de recomendação significativos, capazes de embasar fortemente a adoção de novas condutas. Dessa forma, será possível contribuir para a criação de novos paradigmas e estimular a criação de políticas públicas para o enfrentamento da violência e promoção da saúde.

### Declaração de responsabilidade.

A responsabilidade pelas opiniões expressas neste manuscrito é estritamente dos autores e não reflete necessariamente as opiniões ou políticas da *RPSP/PAJPH* nem da OPAS.
